# Allied Health Students’ Experiences of Telehealth Within Coursework and During Placement: A Survey Study

**DOI:** 10.1177/26924366251388237

**Published:** 2025-10-20

**Authors:** Kate Bridgman, Shane Erickson, Krithika Anil, Adam Bird, Jenny Freeman, Carol McKinstry, Christie Robinson, Sally Abey

**Affiliations:** ^1^ School of Allied Health, Human Services and Sport, La Trobe University, Victoria, Australia.; ^2^ School of Health Professions, University of Plymouth, Plymouth, United Kingdom.; ^3^ School of Health and Biomedical Sciences, RMIT University, Victoria, Australia.; ^4^ La Trobe Rural Health School, Violet Vines Marshman Centre for Rural Health Research, La Trobe University, Victoria, Australia.

**Keywords:** telehealth, allied health students, education, training

## Abstract

**Background:**

Despite growing professional utilization, there is limited understanding of how allied health students are prepared for telehealth practice through academic coursework and placements. This study investigates students’ exposure to telehealth education, their perceptions of preparedness, and the competencies they believe are needed for graduate practice.

**Methods:**

A cross-sectional online survey was conducted between October 2024 and March 2025 across two Australian universities offering 24 allied health courses. The survey, adapted from existing tools and informed by telehealth competency frameworks, collected quantitative and qualitative data on students’ telehealth learning experiences, placement exposure, and perceived competency needs. Descriptive statistics and content analysis were used to analyze and interpret the data.

**Results:**

Of the 108 respondents from 21 disciplines, only 30% reported receiving telehealth education in their coursework, with most learning limited to background knowledge and delivered via online lectures or self-directed modules. Practical skills such as telehealth session setup and communication were less frequently taught, and only 9.3% of students were assessed on telehealth competencies. Placement exposure was similarly limited, with 25% of students engaging in telehealth activities, primarily through observation. Students rated placement support more positively than coursework preparation. Content analysis revealed strong alignment between student-identified graduate competencies and published telehealth frameworks, including technical proficiency, communication, clinical adaptation, and ethical considerations.

**Discussion:**

Findings highlight significant gaps in telehealth education across allied health programs. Students expressed a preference for hands-on, experiential learning and identified a need for structured, competency-based curricula. The lack of assessment and inconsistent integration of telehealth content suggest poor alignment between learning outcomes and educational delivery. Educators should adopt established telehealth competency frameworks and enhance both academic and placement-based training to better prepare students for using telehealth in contemporary practice.

## Introduction

The COVID-19 pandemic required a rapid, universal uptake of telehealth service delivery.^1^ The use of telehealth has remained in many health services due to the proven efficiency, client preference, increased access to service, and the need to continue to practice in COVID-safe ways. The need for skill development and education has been identified as a critical factor in ensuring sustainable telehealth practices.^2^ Although allied health professionals continue to use telehealth in their practice, there are barriers impacting the use of telehealth such as limited infrastructure and therapist confidence and skills.^3^ Allied health students are also increasingly required to engage in telehealth service delivery as part of their placements^4^ and future graduate practice. There is a limited understanding of what preparation and skills are required.

Anil et al.’s^5^ review of allied telehealth competencies identified that less than one-third of allied health disciplines have published telehealth competencies. Those identified in this review related to the skills, knowledge, or behaviors that are required to deliver telehealth consultations, as well as the requirements of a health service or organization to implement telehealth as a service delivery model.^5^ Jacob et al.’s^6^ three-round Delphi study, defining telehealth competencies for health care curriculum, has recently identified 47 telehealth competencies within 12 domains. The domains relate to principles of telehealth; planning and management, assessment, diagnosis, and treatment; suitability of the environment; professionalism; legal considerations; consumer privacy; consumer safety; access and equity; consumer preference; technology and applicability of telehealth. With a growing evidence base to define core competencies, both for practice^5^ and the higher education telehealth curriculum,^6^ there is a recognized need for a systematic approach to core competency integration into allied health education to prepare students for the telehealth experiences they may encounter during placement or working as new graduates.

The current evidence base examining the telehealth experiences of allied health students during placement remains limited.^7,8^ Similarly, the integration of telehealth into undergraduate and postgraduate curricula appears to be underdeveloped, with few high-quality studies detailing its scope or efficacy. Where telehealth education is included, it is commonly delivered through lectures, online activities, and simulation. Content within the curriculum is reported to include understanding technological requirements and implementation, communication and interpersonal skills, understanding the legal and ethical issues, and discipline-specific evidence-based practice. It has been identified that allied health education relating to telehealth competencies occurs in a non-systematic manner, with low-level studies reporting a range of teaching modalities and little assessment.^9^ Due to the limited research evidence to inform allied health curricula development, this study aims to explore allied health students’ exposure to, and perceptions of, telehealth learning experiences, through the following research questions:

1. What current exposure do allied health students have to telehealth service delivery within their coursework and during placement?
2. How are students prepared and supported to develop telehealth skills and knowledge?
3. What telehealth skills and knowledge do allied health students think they should have to practice as new graduate clinicians?

## Method

The methodology for this study was a cross-sectional online survey. Ethics approval for this study was obtained through the La Trobe University Human Research Ethics Committee (HEC24369) and was registered with the RMIT STEM College Human Ethics Advisory Network.

### Participants and recruitment procedure

Participants were students enrolled in one of 24 allied health courses (listed as Supplementary Data S1) at La Trobe University and RMIT. Together, these two universities offer undergraduate and postgraduate allied health courses in predominantly face-to-face, hybrid, and some fully online teaching modes across four metropolitan and four regional campuses in the state of Victoria, Australia. Course coordinators of each allied health course posted an invitation to participate in the study on the online learning management systems for each year level. Students were invited to participate between October 1, 2024, and March 31, 2025. This allowed students to reflect on their coursework learning and placements for the 2024 academic year.

### Survey development and pilot testing

The survey was adapted from Edirippulige et al.,^10^ for use with allied health students instead of medical students. Adaptations were informed by two scoping reviews that have explored allied health student’s experience of telehealth^5,7^ and the extent of telehealth curriculum in undergraduate and postgraduate allied health courses.^11^ The survey was developed by the first author in consultation with the multidisciplinary authorship team who tested the draft questionnaire. This was to establish the clarity and acceptability of the questionnaire items and the face and content validity of the survey questionnaire.^12^ Feedback was sought in relation to ease of completion, wording and potential ambiguity or bias of questions, relevance and length of questionnaire, whether there were any redundant or missing items, and suitability for a diverse range of allied health disciplines and year levels. Using an iterative process, changes were made to the questionnaire in response to the feedback, and after several rounds, the questionnaire was finalized (refer to Supplementary Data S2 for a complete copy of the survey).

### Data analysis

**FIG. 1.**
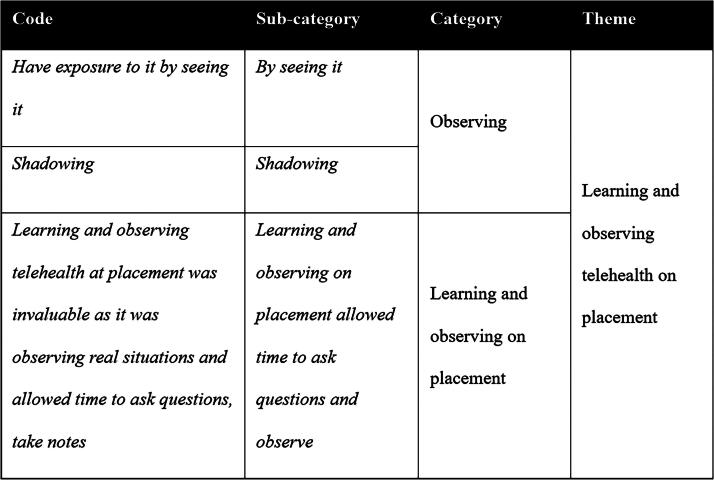
Example content analysis.

Descriptive analysis of quantitative data included frequency counts, means, and medians. Content analysis of short-answer questions followed Graneheim and Lundman’s^13^ content analysis methodology for qualitative text responses. This structured approach began with identifying the codes from the survey responses, then condensing codes to subcategories, and labeling them while ensuring key text and integrity was retained. The subcategories were then grouped into categories and overarching, broader themes as exemplified in Figure 1. Analysis was completed by the first author with support from the second author. Subcategories and categories were reviewed by the full authorship team. This included interrogation of the groupings of codes into subcategories and then alignment of subcategories that were combined to create categories. Finally, the team critically reviewed the illustrative quotes used for each theme to ensure alignment with the provided summary.

## Results

A total of 108 students across 21 disciplines completed the survey. Participants ranged in age from 17 to 58 years (mean = 24.9; median = 21.5) with 85 (78.7%) identifying as female, 20 (18.5%) as male, 1 as nonbinary, and 1 as transgender. One student elected not to disclose their gender. Students were enrolled in bachelor’s or master’s courses at one of two regional campuses (6), four metropolitan campuses (99), or online courses (3). Allied health disciplines and year levels are presented in Table 1. There were no respondents from Exercise Science/Sport and Exercise Science/Exercise Physiology, Podiatry, or Sonography.

**Table 1.** Allied Health Disciplines and Year of Study

Allied health discipline	*n*	Year of study	*n*
Art Therapy	2	Bachelor Year 1	24
Audiology	1	Bachelor Year 2	31
Biomedical Science	13	Bachelor Year 3	20
Chiropractic	1	Bachelor Year 4	11
Diagnostic Imaging	1	Master’s Year 1	11
Dietetics	3	Master’s Year 2	11
Health Information Management	2		
Medical Laboratory Science	5		
Nuclear Medicine	2		
Occupational Therapy	7		
Orthoptics	7		
Osteopathy	4		
Paramedicine	1		
Pharmacy	12		
Physiotherapy	9		
Prosthetics and Orthotics	6		
Psychology	18		
Radiation Therapy	2		
Radiography	4		
Social Work	3		
Speech Pathology	5		

The majority of students (71.2%) reported having prior experience with a health care professional via telephone consultation, and 45.3% had participated in a video-based telehealth consultation as a patient or support person.

### Academic learning

Thirty percent of all students, from across 12 disciplines, reported learning about telehealth in their course. These disciplines included Audiology, Biomedical Science, Dietetics, Health Information Management, Medical Laboratory Science, Occupational Therapy, Orthoptics, Pharmacy, Physiotherapy, Prosthetics and Orthotics, Psychology, and Speech Pathology. Students engaged in a range of topics relating to telehealth knowledge and skills, via a range of learning modalities, as presented in Table 2.

**Table 2.** Telehealth Coursework Content and Learning Modality

Telehealth coursework	*n* ^a^	Learning modality	*n* ^a^
Security and/or safety considerations for a telehealth session	17	Self-directed online learning activities	19
Background or origin of telehealth	16	Live online lectures	15
How to apply assessment and intervention via telehealth	15	Live online workshops	15
Research evidence related to my discipline	13	Case-based/clinical scenarios *without* simulation	4
How to support clients/patients to engage in a telehealth session	12	Case-based/clinical scenarios *with* simulation	4
Communication skills required for a telehealth session	10	Live observation	3
How to select and/or use technology required for a telehealth session	10	Other	0
How to set up a telehealth session	8		
Service management requirements for organizations to deliver telehealth sessions	5		
Other	0		

^a^ Students were able to select all responses that applied to their learning experiences.

While students had the option of selecting more than one topic, none reported engaging in all listed areas. Rather, just under one-third engaged in five or more of the topics, with 41% engaging in just one or two topics. When considering topics in relation to telehealth knowledge compared to skills or application, approximately half of the students had learnt how to undertake assessments in telehealth sessions, while one-third had learnt how to support clients undertaking telehealth sessions. Practical skills of setting up a telehealth session, selecting suitable technology, and communication skills were less often learnt. This did not appear to be related to year level because there were students with exposure to between one and seven or eight topics in each year level of both bachelor and master cohorts.

### Assessment of telehealth learning

Ten students (9.3%) reported being assessed on telehealth within their coursework. These students were from the disciplines of Psychology (3), Prosthetics and Orthotics (2), Occupational Therapy (2), Orthoptics (2), and Biomedical Science (1). They were in year 1, 2, and 4 in Bachelor courses and year 1 and 2 in Master’s courses. The methods of assessment included research essay (9), multiple-choice quiz (7), short-answer test (6), written session plan or report for a case study (5), written reflection (4), and practice demonstration which could be live or video submission involving an oral exam (5). Of these 10 students, 40% indicated that telehealth had not been included in their coursework prior to assessment. Therefore, of the 32 students who reported experience with learning about telehealth in their course, more than 80% reported that they were not assessed on their learning. Of the six students who were exposed to telehealth learning and assessment, alignment between learning practical skills and demonstrating practical skills was only reported by two students.

### Placement experience

Twenty-seven students (25%) indicated that they had undertaken at least one clinical placement that included telehealth. Placement settings included health services (13), private practice (6), community (5), university clinics (4), health education (4), and not-for-profit (1). The 12 disciplines and 6-year levels of these students are detailed in Table 3.

**Table 3.** Allied Health Disciplines and Year of Study for Students with Telehealth Exposure on Clinical Placement

Allied health discipline	*n*	Year of study	*n*
Art Therapy	1	Bachelor Year 1	1
Biomedical Science	4	Bachelor Year 2	3
Dietetics	2	Bachelor Year 3	4
Health Information Management	1	Bachelor Year 4	5
Medical Laboratory Science	1	Master’s Year 1	5
Occupational Therapy	3	Master’s Year 2	7
Orthoptics	1		
Pharmacy	2		
Physiotherapy	5		
Prosthetics and Orthotics	1		
Psychology	5		
Radiography	1		

Students were asked about their experience with observing and using telephone and/or video telehealth and how they were prepared for conducting telehealth sessions. Of the 27 students who had experience using telehealth on placement, two-thirds of students had observed their educator deliver health information or conduct a consultation via telephone, while 44% had the opportunity to conduct a telephone telehealth activity. Twelve (44%) of these students conducted telephone telehealth on placement, three did not have an opportunity to observe their educator, and only half received preparation or support to prepare. Eleven (41%) students had an opportunity to observe their educator using video telehealth during their placement, and seven students conducted a video telehealth session. Only two of the seven students received support or preparation from their educator before using video telehealth. The support and preparation strategies provided for students are detailed in Table 4.

**Table 4.** Telehealth Support and Preparation Strategies

Preparation or support strategy^a^	Telephone	Video
Observation of another clinician	4	1
Review of polices and/or procedures	1	0
Verbal explanation, suggestions, instructions, and/or education	5	2
Feedback on a written plan or session plan	1	1
Opportunity to role-play—with no feedback	0	1
Opportunity to role-play—with feedback	2	1
Opportunity to practise independently with the technology	0	1

^a^ Students received more than one support or preparation strategy.

**FIG. 2.**
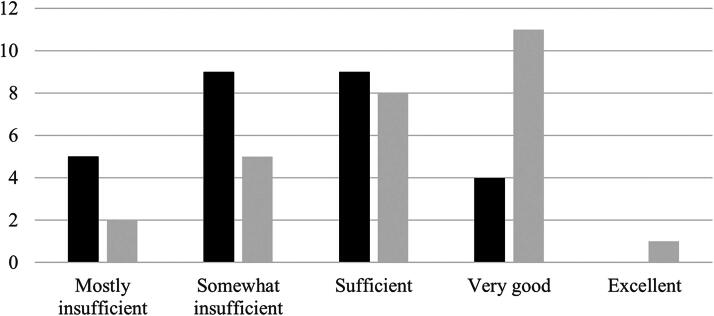
Student description of telehealth preparation and support. 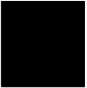 Coursework in preparation for placement; 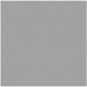 Practice educator during placement.

Students were also asked to rate the telehealth preparation and support provided in their coursework prior to placement and by their practice educator during placement, using a 5-point Likert scale, as shown in Figure 2. The results indicate that students perceived the support during placement to be more adequate than the preparation provided through coursework before placement.

Content analysis and frequency count of student responses in open-ended questions were undertaken related to Likert scale ratings. Overall, 14 students reported not receiving telehealth preparation or education in their coursework: “*little to no experience or education. Given the post-covid climate, would be very beneficial to incorporate into the curriculum*” (Bachelor Year 4 Physiotherapy student). A further 10 students reported that they had received some education relating to telehealth. Topics included “*safety and security for telehealth*” (Bachelor Year 2 Biomedical Science student), “*basic content on telehealth*” (Master’s Year 2 Dietetics student), and “*benefits and risks*” (Master’s Year 2 Occupational Therapy student). The final three students indicated they had received “*information [that] was detailed*” (Master’s Year 1 Psychology student) and “*a lot of preparation methods*” (Master’s Year 1 Physiotherapy student).

Six students identified not being prepared or supported by their practice educator: “*Nil support re: using telehealth. Was told to ‘just do it’*” (Master’s Year 2 Dietetics student), four were provided resources for self-directed learning: “*I have been shown the resources to complete the tasks by myself*” (Master’s Year 2 Psychology student), five were provided an opportunity to observe a telehealth session: “*Just observed. Didn’t actually get taught how*” (Master’s Year 2 Occupational Therapy student) and “*Very good because it was offered and I was able to observe phone call telehealth, some more observations of video telehealth would be even better*” (Master’s Year 2 Art Therapy student), and one student “*got feedback and opportunity to practice before attempting calls*” (Master’s Year 2 Occupational Therapy student).

**FIG. 3.**
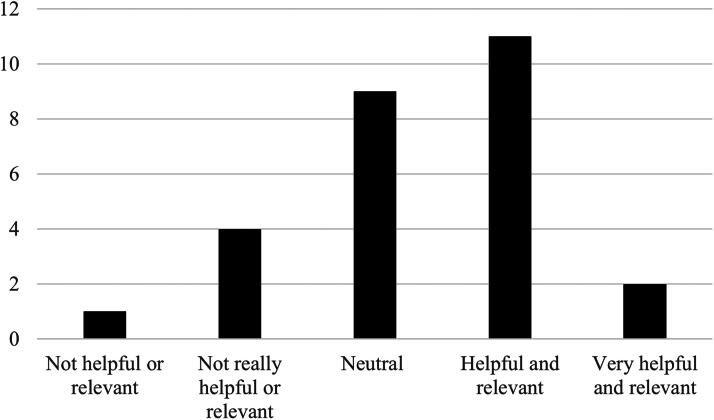
Student rating of placement helpfulness in developing telehealth competencies.

Students were also asked to indicate how helpful their telehealth placement experience was in relation to developing their telehealth competencies as a clinician (Fig. 3). This indicates that 13 students found placement helpful and relevant in developing their telehealth competencies compared to 5 who reported that it was not helpful.

Content analysis and frequency count of student responses were undertaken relating to their Likert scale ratings. Six students indicated that observing a telehealth consultation was helpful, but not enough to develop their competency:

“*Witnessing a Telehealth appointment on placement gave me a little bit of exposure to how Telehealth can be used in clinical practice. But one appointment alone is not sufficient for me to feel confident enough to incorporate Telehealth into my own clinical practice*.” (Master’s Year 1 Physiotherapy student)

Four students identified that practice on placement was helpful: “*I became more comfortable talking over the phone with practice*” (Master’s Year 2 Dietetics student). A further five students shared the telehealth experiences they had, which included telephone use, education for clients, families and carers, and direct clinical activities: “*We could not see exercise moves from the waist down with some people’s cameras, so we had to be very clear with instructions and demonstrate well*” (Master’s Year 1 Physiotherapy student).

### Student preference in telehealth learning modality

Content analysis of student responses to an open-ended question about their preferences for telehealth learning identified one main theme.

Students differ in their preference for telehealth learning modalities, activities, and whether this occurs within coursework or as part of clinical placement. Student preferences for how they would like to learn telehealth knowledge and skills included *lectures*, *workshops*, *tutorials*, *seminars*, *online modules*, and *webinars*. Students identified a variety of learning activities that they would prefer to undertake to learn telehealth skills and knowledge. These ranged from being provided *internet sites* and *a resource bank*, to *role-play*, *practice with peers*, *case-based scenarios*, *simulation*, and *hands-on real-life experience*.

“*‘Practice telehealth consults run during tutorials (if possible) where students can use the software to connect with other students in a ‘mock’ appointment setting. This will help us to get more familiar with using the software.*” (Bachelor Year 1 Psychology student)

“*I would like to do practical tasks to see if my peers and I can communicate over the phone properly. I think this would be really beneficial as sometimes you can have bad experiences*.” (Bachelor Year 1 Pharmacy Student)

Students want to learn from and observe professionals who integrate telehealth into their practice. They would like exposure on placement that includes observation, shadowing an experienced clinician, an opportunity for practice, and receiving support and feedback about their own delivery of telehealth:

“*Learning and observing telehealth at placement was invaluable as it was observing real situations and allowed time to ask questions, take notes and also to have a go with a supervisor present*.” (Master’s Year 2 Art Therapy Student)

“*Observing experienced professionals in telehealth consultations and receiving feedback on my practice would help enhance my skills and confidence*.” (Bachelor Year 2 Biomedicine Student)

Finally, some students indicated that they would like to complete a range of learning activities that span coursework and placement:

“*I would like to learn about telehealth through a combination of interactive online courses that include practical simulations and case studies, as well as webinars led by experienced professionals in the field. Engaging in hands-on workshops would allow me to practise using various telehealth platforms and tools, while mentorship or shadowing opportunities with seasoned telehealth practitioners would provide real-world insights and guidance*.” (Bachelor Year 1 Psychology student)

“*Lectures, live observations through placement and conducting our own Telehealth consultations on placement*.” (Master’s Year 1 Audiology student)

### Student-identified graduate telehealth knowledge and skills

All students who conducted the survey were asked to identify the telehealth skills and knowledge they thought would be required as an allied health graduate. Content analysis of student open-ended responses identified nine themes, as detailed in Table 5.

**Table 5.** Student-Identified Graduate Telehealth Knowledge and Skills

Theme	Descriptor	Illustrative Quotes
Technical proficiency, including platform selection, session setup, and troubleshooting	Having technology skills and knowledge that allow selection and understanding of the specific platform that is being used, being able to set up and conduct a session, and engage in problem-solving and troubleshooting to manage technical difficulties.	“*I believe I will need to have proficient technology skills and a solid understanding of the platform I will be using*” (Bachelor Year 1 Paramedicine student). “*Use the software and troubleshoot technical difficulties within telehealth programs*” (Bachelor Year 1 Psychology student). “*Also, some general knowledge of how to use the technology for setting up*” (Bachelor Year 2 Orthoptics student).
Decision-making about the suitability of telehealth, including limitations and barriers	Being able to determine *how and why to use telehealth*, *what conditions are suitable* while *ensuring safe conduct* that is *within scope*; recognizing *limitations and barriers* to use for specific populations and *considering options for those who cannot access telehealth.*	“*You need to know what can and cannot be managed over a telehealth consult*” (Bachelor Year 3 Pharmacy student). “*Ensuring that I am recognising the barriers that some people might face with Telehealth and ensuring I adapt my approach to meet the diverse needs of the population (e.g., language barriers, cultural preferences, and differing levels of digital literacy)*” (Master’s Year 1 Psychology student).
Client/patient telehealth education and technical support	Being able to educate clients *about the benefits of telehealth, the different means of conducting telehealth*, and *how to best assist clients with telehealth consultations.*	“*… so education regarding the benefits for rural populations could help, as well as time/money saving by completing some subjective assessments over telehealth prior to a home visit etc*” (Master’s Year 1 Physiotherapy student). “*And how to best assist clients with telehealth consultations*” (Bachelor Year 4 Prosthetics and Orthotics student).
To adapt assessment, diagnose accurately, and deliver effective treatment	How to *adapt* and *navigate assessments*, ensuring they are *appropriate* and accurate; how to *adapt clinical interventions for virtual delivering*, ensuring *efficacy of interventions telehealth* including *correctly demonstrate and instruct exercises.*	“*How to do intervention sessions over zoom, especially for clients who are unable to leave their home, or who may need support to come to health services*” (Master’s Year 2 Art Therapy). “*Skills around adapting Ax (assessment) and Tx (treatment)*” (Bachelor Year 3 Speech Pathology).
Clinical knowledge and understanding of clinical outcomes	Discipline-specific clinical knowledge including *knowledge of symptoms* and *clinical outcomes.*	“*Understanding Clinical Outcomes: It’s important to comprehend and interpret clinical outcomes communicated remotely, ensuring proper follow-up and adjustments to care as needed*” (Bachelor Year 2 Pharmacy student).
Communication and interpersonal skills, including cultural competency	How to communicate *in a professional manner, provide information to patients effectively, use appropriate language* while also demonstrating *active listening*, and determine *if the person is actually listening.* Interpersonal skills include *eye contact, understanding body language, maintain attention/engagement*, and *building rapport* while demonstrating *compassion, confidence, patience*, and *empathy. Developing cultural competence to engage diverse populations.*	“*How to communicate with patients over the phone*” (Bachelor Year 4 Radiography student). “*Ability to understand body language, tone, posture, as it’s hard to gauge how someone is responding/listening if you just see them on a video*” (Master’s Year 2 Dietetics student). “*Cultural Sensitivity: Being able to engage with diverse patients and colleagues while maintaining awareness of cultural factors, which can impact communication and understanding in a telehealth setting*” (Bachelor Year 2 Pharmacy student).
Professionalism, staying updated, and interprofessional collaboration	Knowing *how to present via video/phone*, *maintain professional standards*, and stay *updated with telehealth practices and technologies.* Being able to *work with other health care professionals.*	“*Professionalism: Maintain professional standards in a virtual setting*” (Bachelor Year 2 Biomedical Science student). “*Staying updated on the latest telehealth guidelines and best practices will enhance your ability to deliver high-quality care*” (Bachelor Year 1 Occupational Therapy student). “*Capacity for interprofessional collaboration are [is] vital*” (Bachelor Year 1 Psychology student).
Create service engagement and maintain attendance	How to *create engagement* and maintain *attendance*, including *safely transitioning a client from face-to-face to telehealth consults.*	“*How to create engagement with services via telehealth*” (Master’s Year 2 Art Therapy). “*Maintaining rate of attendance*” (Master’s Year 2 Occupational Therapy).
Manage data security, privacy, confidentiality, and client/patient safety	To understand and manage *data security, privacy and confidentiality*, and *legal requirements.* To look for indicators of *coercion or abuse,* and support clients *to manage triggers or emotional regulation* in relation to their environments.	“*The security risks around Telehealth and how to manage the flow of health Information*” (Bachelor Year 4 Health Information Management student). “*Legal and ethical considerations to ensure you are complying with legal requirements regarding client confidentiality, informed consent, and knowing the laws and regulations about providing Telehealth services across state borders*” (Master’s Year 1 Psychology student). “*I feel I have to consider the environment the client is in and how they might manage triggers or emotion regulation*” (Bachelor Year 3 Social Work student).

## Discussion

The recent significant uptake of telehealth-administered allied health services^14^ has been underpinned by research showing telehealth can be effective, efficient, and accessible (e.g., Raymond et al.^15^; Barrett et al.^3^) for service-users and practitioners. Indeed, over 70% of the students surveyed in this study had used telephone, and 45% had used video, for their own personal telehealth care. Tertiary institutions have a responsibility to adequately prepare graduates to enter the workforce, yet little is known about whether this includes exposure to telehealth content and assessment within coursework and clinical placement. Furthermore, it is important to establish whether students perceive any preparation they receive as being adequate.

This cross-sectional online survey of 108 students studying in one of 24 university-based allied health courses, representing 21 disciplines, is the first to capture data from such a breadth of allied health professions. While previous research has reported on the content and assessment embedded in specifically designed telehealth subjects or experiences (see Hui et al.^8^ for a scoping review), no study has used a cross-sectional survey methodology to identify any telehealth curricula being delivered across such a broad suite of allied health courses, including clinical placement experiences.

As found in previous research (i.e., Edirippulige et al.^9^), the students in our study desired the opportunity to develop both their telehealth knowledge and skills. Furthermore, our content analysis of responses regarding the participants’ perceptions of what they need to learn aligns with the important telehealth competencies identified in allied health research and professional documentation. Anil and colleagues’^5^ scoping review of published telehealth competencies identified 11 competency themes grouped into consultation or service domains. When asked what telehealth knowledge or skills allied health graduates require, the themes generated from student responses in our study demonstrated a high degree of congruency with Anil and colleagues’ identified competency themes. Furthermore, there is close alignment with more recently published telehealth core competencies for higher education generated by telehealth experts, clinicians, and educators (Jacob et al.^6^).

Yet, although students understood the telehealth skills they need and are eager to learn them, only one in three reported receiving relevant coursework. For those students who received telehealth content, the breadth of the learning was limited, and less than half reported it to be sufficient for their needs. Approximately 40% of participants had only learnt about one or two of the presented telehealth topics, with no apparent alignment to the year level of the course they studied. Much of the content was reported to focus on background knowledge, that is, the origin of telehealth, and discipline-specific telehealth research evidence. Little content focused on required competencies and behaviors such as the necessary communication skills, how to set up a telehealth session, and selecting and practicing using suitable technologies. Furthermore, the modality of learning frequently did not align with student preferences. Most students wanted to engage in practical activities such as role-play, practice with peers, case-based scenarios, simulation, and hands-on real-life experience. However, the content they received was largely delivered via self-directed online learning activities, online lectures, or workshops.

Assessment of telehealth knowledge and skills was reported to be almost nonexistent. For those students who had been taught telehealth content, the vast majority reported not being assessed on this learning. Concerningly, several students who reported being assessed on their telehealth knowledge did this despite reporting not being taught the necessary content. Most of the limited assessment occurred via essays and quizzes or tests. Very few assessment tasks were reported to assess practical skills. The lack of assessment reflects poor constructive alignment that is inconsistent with the way telehealth curricula is often presented in published research (e.g., Hui et al.^8^; Serve et al.^6^). It is hypothesized that rather than properly planning the alignment of telehealth intended learning outcomes, teaching and learning activities, and assessment tasks, educators are including telehealth content in a more haphazard manner.

Given the limited coursework preparation and assessment reported by the students in our study, it is unlikely that many would be adequately prepared to use telehealth in clinical placements. Clinical placements can give students the opportunity to learn about telehealth, and the surveyed students wanted to observe telehealth in action from experienced clinicians. However, exposure to telehealth in clinical placements remains limited, with only one in four students reportedly engaging in telehealth in this context. These experiences primarily occurred in health services, with several students also engaging in telehealth in private practice and community health settings. In contrast to their academic coursework, more students favorably reported on the telehealth preparation and support they received from practice educators. This is unsurprising given the beneficial role allied health practice educators are reported to play in clinical placements more broadly (e.g., Hall et al.^16^). However, it should be noted that little is known about the telehealth preparedness, knowledge, and skills that allied health practice educators possess to guide students on clinical placements. Overall, while students recognize the value of telehealth and are eager to build their skills, inconsistent and often insufficient coursework and placement experiences suggest that it is likely that many are not adequately prepared for telehealth practice as future allied health professionals.

## Recommendations

While telehealth has been used by allied health clinicians for many years, its rapid uptake during the COVID years and beyond appears to have not yet become embedded in curricula by allied health educators. Students in a wide range of allied health courses at two major universities experienced varied levels of exposure to the concepts and skills necessary for telehealth implementation. It should be acknowledged that there is limited published literature to guide the development of this curriculum (Hui et al.^8^; Serve et al.^7^). However, educators have a responsibility to recognize the importance of telehealth in the curriculum and ensure constructive alignment when educating students.

The findings of this research, alongside the current conclusions from the literature, should encourage allied health educators to closely review the content, modality, and assessment of telehealth in their courses. At a minimum, educators should draw upon published telehealth competency themes identified from the literature (e.g., Anil et al.^5^) and telehealth competency frameworks (e.g., Jacobs et al.^6^) to ensure these are sufficiently taught and assessed through both academic coursework and clinical placement experiences. Furthermore, educators should seek to evaluate and publish their telehealth curricula to contribute to the limited published literature regarding undergraduate and postgraduate level training.

## Limitations

This research is a stepping stone toward larger, more representative studies. The student participants in our study attended one of only two tertiary institutions. Moreover, many of the included courses were represented by a small number of students. The nature of the recruitment method may also mean that respondents had a particular interest in telehealth education or were otherwise different in their experiences of telehealth education than other students in their courses. Future research should seek to evaluate a larger sample of students from a broader range of institutions.

## Conclusion

This cross-sectional survey study reports student experiences of telehealth learning across 21 allied health disciplines. There are several key conclusions: (1) students are not satisfied with the current telehealth education and training they receive, (2) students prefer an experiential approach with more active participation to their learning rather than didactic methods such as lectures, and (3) students are frequently not assessed on their telehealth competencies, resulting in a gap in understanding the key education and training needs to ensure high-quality telehealth outcomes in practice. Finally, there is a gap in our knowledge regarding the attitudes and experiences of academic and practice educators in training students to use telehealth. To help close this identified gap, efforts should include establishing the methods educators use to design and implement telehealth education in both the classroom and clinical placements.
