# Supplementary material for: Allied Health Students’ Experiences of Telehealth Within Coursework and During Placement: A Survey Study
Source: Telemed Rep. 2025 Oct 20;6(1):352–62. doi: 10.1177/26924366251388237 (PMC12725421; doi:10.1177/26924366251388237)
Supplement: Supplementary Data S1 [file 26924366251388237_supplementary_data_s1.docx]

Supplementary Information - Allied Health Courses

1. Art Therapy
2. Audiology
3. Biomedical Science
4. Chiropractic
5. Diagnostic Imaging Medical Physics
6. Dietetics
7. Exercise Science/Sport and Exercise Science/Exercise Physiology
8. Health information management
9. Medical Laboratory Science
10. Nuclear Medicine
11. Occupational Therapy
12. Orthoptics
13. Osteopathy
14. Paramedicine
15. Pharmacy
16. Physiotherapy
17. Podiatry
18. Prosthetics & Orthotics
19. Psychology
20. Radiation Therapy
21. Radiography
22. Social Work
23. Sonography
24. Speech Pathology
