# Supplementary material for: Allied Health Students’ Experiences of Telehealth Within Coursework and During Placement: A Survey Study
Source: Telemed Rep. 2025 Oct 20;6(1):352–62. doi: 10.1177/26924366251388237 (PMC12725421; doi:10.1177/26924366251388237)
Supplement: Supplementary Data S2 [file 26924366251388237_supplementary_data_s2.docx]

Allied health students' experiences of telehealth within coursework and during placement

This survey is designed to better understand:

- your experiences of learning about telehealth within your course
- your experiences with telehealth on clinical/practice/fieldwork placements

Telehealth refers to "the delivery of healthcare at a distance using information communications technology (ICT). Telehealth is the modality used to connect and provide care - it connects clinicians or any other person(s) responsible for providing care to patient/s and carer/s. It can be used for the purposes of assessment, intervention, consultation, education and/or supervision" (NSW Government, 2019). Examples may include - telephone or video health appointments with a GP or other health practitioner.

**Section 1: About You**

**In this section you will be asked questions about you and the telehealth experiences you may have had before you started your allied health course.**

**Telehealth refers to "the delivery of healthcare at a distance using information communications technology (ICT). Telehealth is the modality used to connect and provide care**

**- it connects clinicians or any other person(s) responsible for providing care to patient/s and carer/s. It can be used for the purposes of assessment, intervention, consultation, education and/or supervision" (NSW Government, 2019). Examples may include - telephone or video**

**health appointments with a GP or other health practitioner.**

How old are you? (Answer in years - i.e. 19, 22, 32).

To which gender do you most identify? Female Male Transgender

Non-binary/non-conforming Prefer not to respond


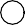

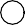

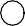

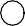

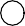


Have you previously engaged in telehealth No

consultations with a healthcare practitioner via Yes telephone in your personal life?


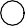

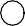


If yes, please detail what you engaged in (select all
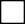
 As a client/patient/service user? that apply):
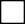
 Supporting a family member/friend


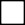
 Other - please provide details

Have you previously engaged in telehealth No

consultations via video with a healthcare practitioner Yes in your personal life?


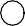

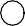


If yes, please detail what you engaged in (select all
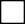
 As a client/patient/service user? that apply):
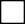
 Supporting a family member/friend


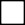
 Other - please provide details

Before commencing this course, have you had previous
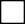
 No

professional experience delivering telehealth
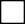
 Yes via telephone

consultations?
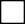
 Yes via video

Have you engaged in professional learning relating to No

telehealth before commencing this course? Yes


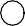

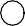


**Section 2: Your current enrolment**

**In this section you will be asked questions about the university course you are currently enrolled in. Please answer the following question based on 2024. Ie if you completed year 2 in 2024, select year 2.**

Which University are you currently enrolled? La Trobe University RMIT


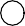

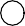


Which La Trobe campus are you enrolled at? Bundoora Bendigo Shepparton Mildura

Albury-Wodonga Melbourne City Online


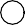

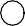

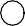

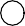

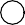

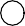

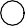

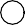

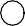


Which RMIT campus are you enrolled at? Bundoora Melbourne City

Which year are you currently studying? Bachelor Year 1 Bachelor Year 2

Bachelor Year 3


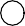

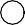

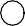

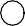

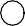

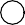


Bachelor Year 4

Masters Year 1

Masters Year 2

Which discipline are you enrolled in? Art Therapy Audiology Dietetics

Health information management Occupational Therapy


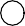

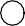

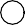

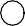

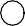

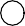

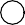

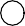

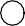

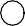

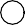

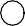

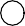

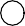

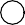

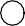


Oral Health/Dentistry Orthoptics Paramedicine Pharmacy Physiotherapy Podiatry

Prosthetics & Orthotics Psychology

Social Work Speech Pathology

Exercise Science/Sport and Exercise Science/Exercise Physiology

Which discipline are you enrolled in? Biomedical Science Chiropractic

Diagnostic Imaging Medical Physics Medical Laboratory Science Nuclear Medicine


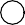

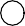

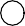

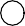

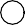

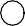

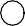

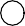

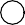

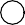

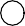

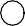

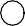

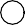


Osteopathy

Radiation Oncology Medical Physics Radiation Therapy

Radiography Pharmacy Physiotherapy Psychology Sonography Social Work

**Section 3: Exposure to telehealth within your current program or coursework up until Dec 31, 2024.**

**In this section you will be asked questions about your exposure to telehealth during your course up until Dec 31, 2024. For this section we are interested in your academic learning about telehealth (i.e. in classes, workshops or assessments). This does not include placements.**

**Telehealth refers to "the delivery of healthcare at a distance using information communications technology (ICT). Telehealth is the modality used to connect and provide care**

**- it connects clinicians or any other person(s) responsible for providing care to patient/s and carer/s. It can be used for the purposes of assessment, intervention, consultation, education and/or supervision" (NSW Government, 2019). Examples may include - telephone or video**

**health appointments with a GP or other health practitioner.**

Have you had previous experience learning about No


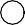

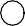


telehealth in your in your academic subjects? Yes

If yes, what did you learn? (select all that apply)
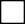
 Background or origin of telehealth


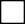
 Research evidence related to my discipline


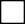
 Communication skills required for a telehealth session


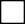
 How to set up a telehealth session


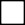
 How to support clients/patients to engage in a telehealth session


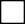
 How to select and/or use technology required for a telehealth session


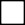
 How to apply assessment and intervention via telehealth


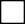
 security and/or safety considerations for a telehealth session


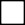
 Service management requirements for organisations to deliver telehealth sessions


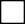
 Other - please provide details

How did you engage in this learning? (select all that
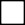
 Self-directed online learning activities apply):
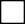
 Live online lectures


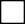
 Live online workshops


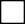
 In person/ on campus lectures


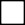
 Case based / clinical scenarios without simulation
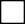
 Case based / clinical scenarios with simulation


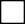
 Live observation


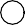

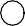


Have you had previous experience of being assessed in No telehealth in your academic subjects? Yes

If yes, how were you assessed? (select all that apply)
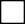
 Multiple choice quiz


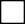
 Short answer test


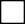
 Written session plan or report for a case study
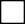
 Written reflection


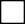
 Research essay


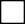
 Practice/demonstration (live or video submission including oral exam)


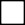
 Other - please provide details

**Section 4: Preparation and exposure to telehealth during your clinical/practice/fieldwork placements up until Dec 31, 2024.**

**In this section you will be asked questions about your exposure to telehealth during your placement/s up until Dec 31, 2024 and how you were prepared for this.**

**Telehealth refers to "the delivery of healthcare at a distance using information communications technology (ICT). Telehealth is the modality used to connect and provide care**

**- it connects clinicians or any other person(s) responsible for providing care to patient/s and carer/s. It can be used for the purposes of assessment, intervention, consultation, education and/or supervision" (NSW Government, 2019). Examples may include - telephone or video**

**health appointments with a GP or other health practitioner.**

Have you completed at least one placement (either in No


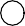

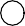


an internal University clinic or external placement) Yes that has included telehealth?

If yes, please indicate all of the placement settings
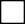
 University clinic

that you have been exposed to telehealth (select all
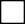
 External placement - health services that apply):
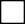
 External placement - education

External placement - private practice External placement - community

External placement - not-for-profit

External placement - other please describe Other - please provide details

Which statement best describes your experience to date I have observed my educator deliver health

with observing telephone telehealth on your information or conduct a consultation via telephone

placement/s? I have not observed my educator deliver health information or conduct a consultation via telephone

Which statement best describes your experience to date I have delivered health information or conducted a with using telephone telehealth on your placement/s? consultation via telephone

I have not delivered health information or conducted a consultation via telephone

Did you receive preparation or any support before you Yes used the telephone on placement? No

Which statements best describe the preparation for the Observation of another clinician telephone telehealth activity you participated in Review of polices and/or procedures

during any placement where you were exposed to Verbal explanation, suggestions, instructions telephone telehealth? (select all that apply): and/or education

Feedback on a written plan or session plan Opportunity to role play - with no feedback Opportunity to role play - with feedback

Opportunity to practise independently with the technology

Other - please provide details

Which statements best describe your experience to date I have observed my educator deliver health with observing video telehealth on your placement/s? information or conduct a consultation via

telehealth that used a camera to allow video connection

I have not observed my educator deliver health information or conduct a consultation via telehealth that used a camera to allow video connection

Which statement best describes your experience to date I have delivered health information or conducted a with using video telehealth on your placement/s? consultation via telehealth that used a camera to (select all that apply): allow video connection

I have not delivered health information or conducted a consultation via telehealth that used a camera to allow video connection

Did you receive preparation or any support before you Yes used video telehealth on placement? No

Which statement best describes the preparation for the Observation of another clinician video/camera telehealth activity you participated in Review of polices and/or procedure

during any placement where you were exposed to Verbal explanation, suggestions, instructions video/camera telehealth? (select all that apply): and/or education

Feedback on a written plan or session plan Opportunity to role play - with no feedback Opportunity to role play - with feedback

Opportunity to practise independently with the technology

Other - please provide details

How would you describe the telehealth preparation and 1 = mostly insufficient support provided by your coursework before starting 2 = somewhat insufficient placement? 3 = sufficient

4 = very good

5 = excellent

Provide a reason for your response:

How would you describe your telehealth experience on 1 = not helpful or relevant placement in relation to developing your telehealth 2 = not really helpful or relevant competency as a clinician? 3 = neutral

4 = helpful and relevant

5 = very helpful and relevant

Provide a reason for your response:

How would you describe the preparation and support 1 = mostly insufficient provided by your practice educator during placement? 2 = somewhat insufficient

3 = sufficient

4 = very good

5 = excellent

Provide a reason for your response:

**Section 5: Future exposure to telehealth**

**In this section you will be asked questions about your expectations for learning experiences related to telehealth.**

What telehealth knowledge or skills do you think you need as an allied health graduate?

How would you like to learn about telehealth to increase your knowledge and skills?
